# CANTARE: finding and visualizing network-based multi-omic predictive models

**DOI:** 10.1186/s12859-021-04016-8

**Published:** 2021-02-19

**Authors:** Janet C. Siebert, Martine Saint-Cyr, Sarah J. Borengasser, Brandie D. Wagner, Catherine A. Lozupone, Carsten Görg

**Affiliations:** 1 Department of Medicine, University of Colorado Anschutz Medical Campus, Aurora, CO USA; 2 Department of Pediatrics, University of Colorado Anschutz Medical Campus, Aurora, CO USA; 3 Department of Biostatistics and Informatics, Colorado School of Public Health, Aurora, CO USA

**Keywords:** Multi-omic, Predictive model, Microbiome, Metabolome, Metagenome, IBD

## Abstract

**Background:**

One goal of multi-omic studies is to identify interpretable predictive models for outcomes of interest, with analytes drawn from multiple omes. Such findings could support refined biological insight and hypothesis generation. However, standard analytical approaches are not designed to be “ome aware.” Thus, some researchers analyze data from one ome at a time, and then combine predictions across omes. Others resort to correlation studies, cataloging pairwise relationships, but lacking an obvious approach for cohesive and interpretable summaries of these catalogs.

**Methods:**

We present a novel workflow for building predictive regression models from network neighborhoods in multi-omic networks. First, we generate pairwise regression models across all pairs of analytes from all omes, encoding the resulting “top table” of relationships in a network. Then, we build predictive logistic regression models using the analytes in network neighborhoods of interest. We call this method CANTARE (Consolidated Analysis of Network Topology And Regression Elements).

**Results:**

We applied CANTARE to previously published data from healthy controls and patients with inflammatory bowel disease (IBD) consisting of three omes: gut microbiome, metabolomics, and microbial-derived enzymes. We identified 8 unique predictive models with AUC > 0.90. The number of predictors in these models ranged from 3 to 13. We compare the results of CANTARE to random forests and elastic-net penalized regressions, analyzing AUC, predictions, and predictors. CANTARE AUC values were competitive with those generated by random forests and  penalized regressions. The top 3 CANTARE models had a greater dynamic range of predicted probabilities than did random forests and penalized regressions (p-value = 1.35 × 10^–5^). CANTARE models were significantly more likely to prioritize predictors from multiple omes than were the alternatives (p-value = 0.005). We also showed that predictive models from a network based on pairwise models with an interaction term for IBD have higher AUC than predictive models built from a correlation network (p-value = 0.016). R scripts and a CANTARE User’s Guide are available at https://sourceforge.net/projects/cytomelodics/files/CANTARE/.

**Conclusion:**

CANTARE offers a flexible approach for building parsimonious, interpretable multi-omic models. These models yield quantitative and directional effect sizes for predictors and support the generation of hypotheses for follow-up investigation.

## Background

Multi-omic approaches in human studies offer exciting opportunities to better understand human health and disease. For example, Ghaemi et al. characterized changes across the transcriptome, proteome, metabolome, microbiome, and immunome during pregnancy [1]. Alfano et al. studied the methylome, the transcriptome, the metabolome and a set of inflammatory proteins to identify relationships with birth weight [2]. Franzosa et al. analyzed the gut microbiome, microbial metagenome, and metabolome in inflammatory bowel disease (IBD) which includes Crohn’s disease and ulcerative colitis [3]. However, analyzing these high dimensional and complex data sets to identify and visualize tractable multi-omic patterns remains a challenge.

Some researchers analyze data from one ome at a time, and then combine predictions across omes, perhaps using a weighted average or other ensemble [1, 4, 5]. This approach is sometimes called late integration [6]. One disadvantage of the late integration approach is that interactions of features across omes are difficult to detect [6]. This is particularly problematic for multi-omic studies of host-microbe interaction [7, 8]. Other researchers resort to correlation studies, cataloging numerous pairwise relationships [2, 9–13], often presented visually as heatmaps [3, 10, 12, 13]. Promising analytes from one ome (e.g.transcripts or metabolites) can be further characterized with pathway analysis [2] or functional enrichment [13]. However, these correlation studies may not account for differing relationships by disease state.

One goal of multi-omic studies is to generate hypotheses for follow-up experiments. For example, microbial metabolites differentially expressed in Crohn’s disease have been evaluated for their ability to modulate cytokine profiles in CD4 + T cells from healthy human blood [14]. Various bacterial lysates, or whole fecal bacterial communities, have been used to stimulate human mononuclear cells, with production of anti-inflammatory cytokines and induction of regulatory T cells then assessed [15, 16]. Similarly, intraepithelial cells have been isolated from gut biopsies of patients with IBD and healthy controls, and subjected to ex-vivo stimulation to identify differences in cytokine production [17].

To facilitate biological insight and improve hypothesis generation from these types of experiments, analytical approaches are needed that yield interpretable models from a handful of multi-omic analytes. Furthermore, the ability to visualize individual-level details in the context of such models supports interpretation and the identification of sub-phenotypes. These details provide a way to vet the models and to appreciate the inherent variability of human participants that may otherwise be difficult to detect based only on ranked lists of statistics. In previous work, we developed VOLARE (Visual analysis Of LineAr Regression Elements) to demonstrate the importance of visualizing pairwise relationships across analytes from different omes [18]. In that work, we summarized a “top table” of pairwise relationships in a VOLARE network (hereafter, Vnet), and supported interactive visualization of the underlying regression models. We applied VOLARE to 3 case studies, all of which were limited to two omes. While some interesting cross-omic relationships were identified through interactive exploration, VOLARE lacked a deterministic method for identifying quantitative multi-omic patterns. In the present work, we apply VOLARE to a three-ome study, and leverage network topology and regression techniques to build families of multi-omic predictive models. We call this new approach CANTARE: Consolidated Analysis of Network Topology and Regression Elements. Here we detail the CANTARE workflow and apply CANTARE in an IBD case study to predict a binary outcome, IBD (person with IBD or healthy control). We use a previously published data set that consists of gut microbiome, metabolomics, and microbial-derived enzymes [3].

As part of our methodological contribution, we analyze key CANTARE configuration parameters, comparing results from pairwise regressions with and without an interaction term, and results from predictive models based on network neighborhoods of order 1, 2, and 3. We also compare CANTARE to random forests [19] and elastic net penalized regressions [20]. Random forests [21–23] and elastic net [24–26] are mature methods commonly used in biomedical research. Like CANTARE, random forests, and elastic net support both continuous and binary outcomes. In the case of binary outcomes, both random forests and elastic net yield an overall measure of model performance (area under the curve, AUC) and sample-level predicted probabilities. Elastic net performs feature selection, thereby yielding a list of predictors. Random forests include measures of variable importance, though they do not perform variable selection per se. Thus, we are able to compare CANTARE, random forests, and penalized regressions using model performance, predicted probabilities, and selected/important predictors.

Our method offers several advantages. We leverage network neighborhoods for feature selection, generating interpretable, predictive multi-omic models. These models support continuous or binary outcomes. We account for differing relationships by group in our pairwise cross-omic regressions. We provide individual-level visualization of both cross-omic regressions and multi-variable predictive models. Importantly, although the regression framework yields so-called predictive models, we use these models to support multi-omic insight.

## Methods

### Workflow

**Fig. 1:**
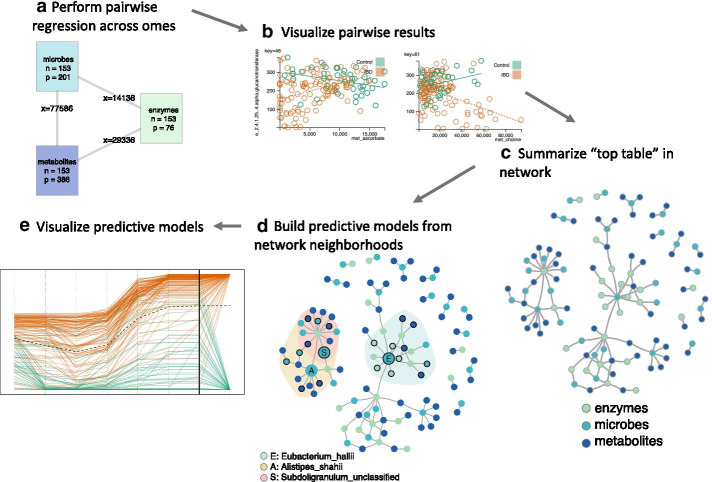
VOLARE-CANTARE workflow. **a**, **b**, and **c **are VOLARE steps. Using the resulting VOLARE network (**c**) as input, **d** and **e** are CANTARE steps. **a** Perform pairwise regressions across all analytes in all omes; n is the number of study participants in each ome, p is the number of predictors in each ome, and x is the number of pairwise regressions. **b** Visualize pairwise results, assessing credibility of underlying detail. Each circle represents one participant. The solid green line represents the fitted regression between the two analytes for controls. The dotted orange line represents the fitted regression for people with IBD. **c** Summarize the “top table” of promising results for each pair of omes in a single network. Each node represents an analyte; each edge represents one relationship from the top table. **d** Build predictive models from network neighborhoods. Shaded regions indicate models created from neighborhoods of order 2, centered on the indicated microbe. Nodes with black borders are included as predictors. **e** Visualize predictive models. The cumulative fit graph displays predicted probability by accumulating the contribution of one predictor at a time. Each path represents values for one study participant (green = control, orange = IBD)

The VOLARE-CANTARE workflow is designed to support hypothesis generation in systems biology studies that include multi-omic data sets. VOLARE generates pairwise regressions across all pairs of omes (Fig. 1a), supports interactive visualization of the underlying pairwise regression models (Fig. 1b), and summarizes the resulting multi-omic “top table” in a network (Fig. 1c), as previously described [18]. Given this network, CANTARE identifies network neighborhoods of interest and builds predictive models from these neighborhoods, using standard regression techniques (Fig. 1d). These models can be visualized with a cumulative fit plot (Fig. 1e). The underlying workflow supports different regression specifications for both the pairwise cross-omic regressions and predictive models based on network neighborhood. In other work [27], we used baseline omic analytes (microbes, DNA methylation sites, and metabolites) to model the changes in clinical outcomes over the course of a short-term behavioral weight loss intervention. The outcomes, which were continuous, were also nodes in the network. In this work, we used network neighborhoods of the microbes to predict a binary outcome, IBD group. IBD group was not a node in the network, but was included in the pairwise regressions with an interaction term, allowing for a different linear relationship between control and IBD samples (Fig. 1b).

### Implementation

The CANTARE workflow is implemented as a customizable sequence of R scripts. It uses the JSON file generated by VOLARE (implementation, R scripts, and documentation, available at https://sourceforge.net/projects/cytomelodics/files/VOLARE/) to initialize the network context; identify network neighborhoods; build predictive models, recording both model summary statistics and details of the predictors; and generate cumulative fit plots and effect size tables from these models. R scripts and a CANTARE User’s Guide are available at https://sourceforge.net/projects/cytomelodics/files/CANTARE/).

### Data set

We apply CANTARE to previously published data from healthy controls and patients with IBD consisting of three omes: gut microbiome, metabolomics, and microbial-derived enzymes. Sample and data processing methods were described in detail in Franzosa et al. [3]. Species-level relative abundance data was derived from shotgun metagenomic data which were taxonomically profiled using MetaPhlAn2 [28]. Only species with greater than 0.1% relative abundance in at least 5 samples were reported. Per-sample gene abundances were normalized to parts-per-million (ppm) and further summed according to Enzyme Commission (EC) number. Like Franzosa, we refer to these as enzymes. Metabolites were measured using both positive and negative ion mode LC/MS, and reported as ppm. From these data, we created a multi-omic data set consisting of all 201 microbial species, 386 metabolites mapped to standards, and expression levels for 76 enzymes. Where multiple metabolite clusters mapped to the same standard, the metabolite with the highest CV (coefficient of variation) was selected. Enzymes were filtered by mean and variance, including only those with mean > 100 and variance > 1000, plus the 5 enzymes discussed in Fig. 5 of Franzosa et al. [3]. We included these 5 enzymes so that we could compare some of our detailed results with those reported by Franzosa. We limited our analysis to the 153 participants for which there were fecal calprotectin results. The multi-omic data we analyzed is in Additional file 1.

### Regression network and predictive models

To build a network of multi-omic relationships that differed by IBD group, we fit a linear model to each pair of analytes across each pair of assays, with an interaction term for IBD group:, e.g.

$$\begin{gathered} for \, i \, in \, all \, enzymes \hfill \\ \;\;\;for \, j \, in \, all \, metabolites \hfill \\ \;\;\;\;\;\;\;\;fit:enzyme(i)\,\sim\,\beta_{0} \, + \,\beta_{1} \, \times \,IBD\, + \,\beta_{2} \, \times \,metabolite\left( j \right)\, + \,\beta_{3} \, \times \,IBD \, x \, metabolite(j). \hfill \\ \end{gathered}$$

To eliminate relationships potentially driven by outliers, we removed models with a maximum DFFITS value ≥ 4. (DFFITS is the standardized difference in fit—the number of standard deviations by which an observation’s estimate changes, when the model is built without that observation.) We also removed relationships based on microbes with non-zero values in fewer than 10% of the samples, leaving in a total of 73,405 pairwise results. We then created a Vnet from the 35 models with the smallest p-values for the interaction term (*β*_3_) from each assay pair, subject to p-value < 0.05 (Fig. 1b).

From this Vnet, we identified the network neighborhoods of order 2 (immediate neighbors, and their neighbors) for each microbe. For each such neighborhood with at least 4 nodes, we built a predictive model for IBD using logistic regression (R method glm with family = binomial) (Fig. 1d). We included age and fecal calprotectin as predictors, due to their clinical relevance. Fecal calprotectin, which can vary by age group, is a reliable marker of intestinal inflammation with good clinical sensitivity for IBD [29]. We treated this as the full model, then performed backward selection with Akaike information criterion (AIC) as the selection criterion  to generate a reduced model. We refit the reduced model using the lrm method in the rms package (which uses maximum likelihood estimation) so that we could access the specialized model diagnostics in that package.

We generated cumulative fit plots by multiplying the regression model matrix by the vector of estimated coefficients. This resulted in a “fit matrix” with one row per person, and columns representing the contribution of each predictor (including the intercept) to the predicted outcome. We then sorted the non-intercept columns based on interquartile range (IQR) effect size, low to high. If the first quartile equaled the third quartile, the effect was expressed in terms of overall range. To create a cumulative fit matrix, we first added the intercept to the column with the smallest effect size to create a single column. For all subsequent columns, we summed the values for all previous columns of the sorted fit matrix. We converted these log-odds estimates to probabilities, and plotted the resulting values, with one path per person (Fig. 1e).

### Comparisons to random forests and penalized regressions

Using two different data sets (all multi-omic data, plus age and fecal calprotectin; and the predictors in the Vnet, plus age and fecal calprotectin), we generated random forests to predict IBD group. We used the R package randomForest, with default parameters (number of candidate variables at each split mtry = sqrt(p), number of trees = 500). The most important variables were those with the greatest mean decrease in the Gini index. Using the same 2 data sets, we fit penalized regressions to predict IBD group using the cv.glment method in the R package glmnet, (alpha = 0.5, number of cross-validation folds = 5, loss function for cross-validation = deviance, and family = binomial).

## Results

### Case study

#### Disease background

IBD, which includes Crohn’s disease (CD) and ulcerative colitis (UC), is an inflammatory disorder of the gastrointestinal (GI) tract, resulting from the complex interactions between genetic make-up, microbiome composition, environmental factors, and mucosal immune response [30]. In the last 10 years, the prevalence of IBD in adult and pediatric patients alike has been steadily increasing worldwide [31]. Though the exact mechanisms underlying the disease pathogenesis are not fully understood, recent studies have found a number of environmental factors including diet, medications, and the gut microbiota that can trigger an overactive mucosal immune response in the host, and have been linked to increasing IBD prevalence [32]. Thus, IBD is a multifactorial disease and complex in its management approach.

The current diagnostic method for IBD consists of a combination of a detailed history assessment, physical and laboratory examination, esophagogastroduodenoscopy, ileo-colonoscopy combined with histology, and imaging of the small bowel [33–35]. Treatment strategies for IBD often entail the usage of pharmaceutical products with long-term effectiveness. However, not all patients respond to and can sustain treatment with these drugs, which have various side effects [36].

#### Data set and analysis workflow

Franzosa et al. characterized differences in microbiome-metabolome correlations in IBD as compared to healthy controls, suggesting that some of these relationships included diagnostic and therapeutic targets [3]. We hypothesized the same data could be used to generate novel and interpretable multi-omic predictive models that would support better understanding of the IBD pathogenesis and contribute to a more comprehensive and precision medicine model for IBD management. Starting with data for 153 participants (42 control, 111 IBD) consisting of microbiome relative abundance (p, the number of predictors = 201), metabolites (p = 386), and microbe associated enzymes (p = 76), we performed pairwise regressions across the omes (Fig. 1a, b). We filtered results to the top 35 per assay pair, from which we created the Vnet. As a heuristic, we include around 100 results in the network, with at least 25 per assay pair. Since microbes are producers and consumers of metabolites and the source of the metagenome, they are central to this data. Furthermore, they have been widely studied in IBD [14, 17, 21, 23, 37, 38]. Thus, we used the network neighborhood of order 2 (a node’s neighbors, and their neighbors) of each microbe to generate predictive models for IBD, limiting model building to the 20 network neighborhoods with at least 4 nodes. The omic analytes included in the top three predictive models, based on area under the curve (AUC), are highlighted in Fig. 1d. The “Subdoligranulum” neighborhood is a subset of the “A. shahii” neighborhood, while the “E. hallii” neighborhood is in a separate subnetwork. Hereafter, we refer to these neighborhoods and models as E, A, and S, representing the seed nodes of *Eubacterium_hallii*, *Alistipes_shahii*, and *Subdoligranulum_unclassified* respectively.

**Fig. 2:**
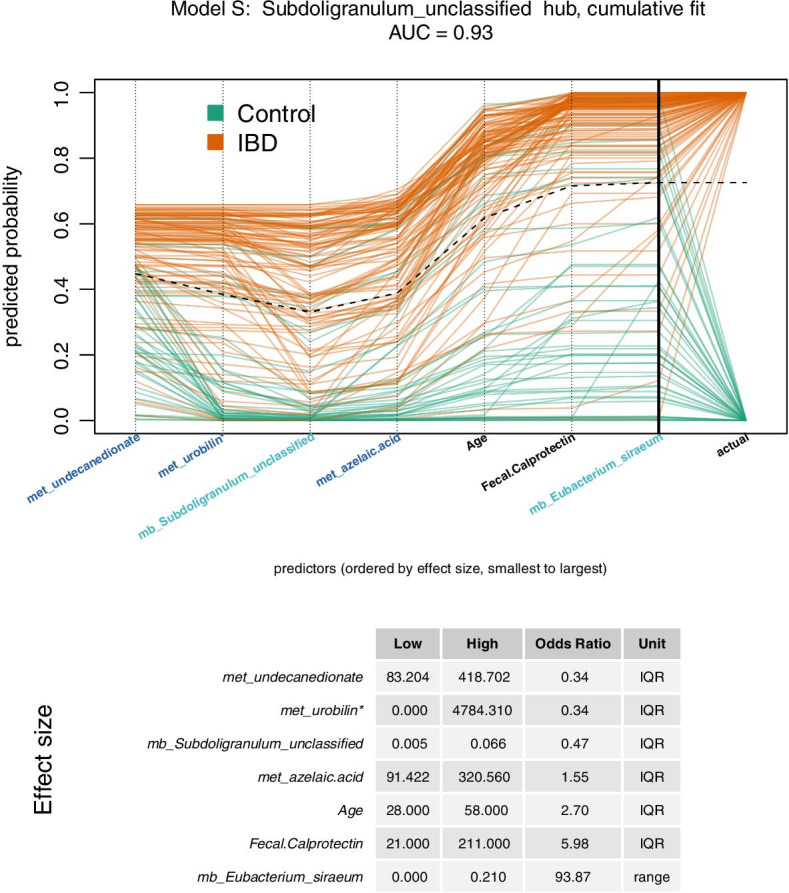
Cumulative fit for Model S. This model was created from the *Subdoligranulum* hub. It has an AUC of 0.93 and the fewest predictors (7) of the top 3 models. The cumulative fit graph displays predicted probability by accumulating the contribution of one predictor at a time. A dotted black “trend line” connects the mean cumulative value at each predictor. Omic predictors are prefaced by met or mb, representing metabolite or microbe respectively. Each orange path represents one study participant with IBD, while each green path represents one control participant. The intersection of the paths and the bold black vertical line represents predicted probability for each person, while the final points of the paths indicate the actual class. The predictors are ordered by effect size, low to high. The intercept is not shown. An isomer of urobilin is indicated by “urobilin*”. Effect sizes for each predictor are expressed as the odds ratio for IBD for either interquartile range (IQR) or overall range. If the first quartile equals the third quartile, the odds ratio is expressed in terms of overall range. The IQR effect size is the change in response associated with a change in a predictor from the first quartile (Q1, 25th percentile) to the third quartile (Q3, 75th percentile), which includes 50% of the data values

**Fig. 3:**
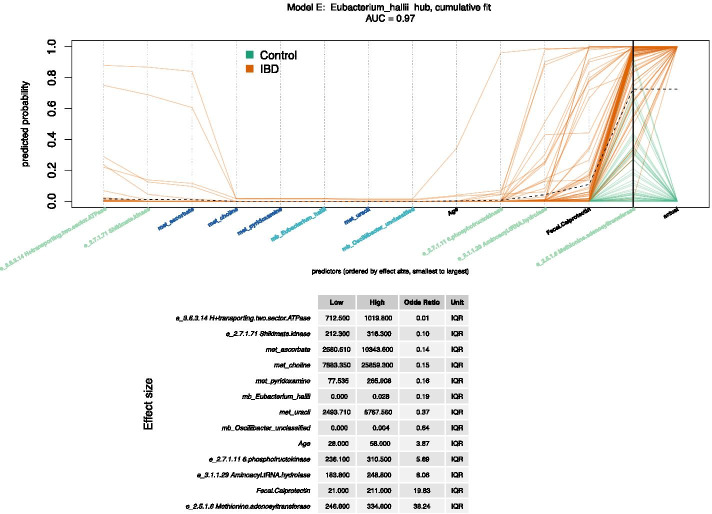
Cumulative fit for Model E. This model was created from the *E. hallii* hub. It has the best AUC (0.97) of the top 3 models and the most predictors (13). The cumulative fit graph displays predicted probability by accumulating the contribution of one predictor at a time. A dotted black “trend line” connects the mean cumulative value at each predictor. Omic predictors are prefaced by e, met, or mb, representing enzyme, metabolite, or microbe respectively. Each orange path represents one study participant with IBD, while each green path represents one control participant. The intersection of the paths and the bold black vertical line represents predicted probability for each person, while the final points of the paths indicate the actual class. The predictors are ordered by IQR effect size, low to high. The intercept is not shown. Effect sizes for each predictor are expressed as the odds ratio for IBD for interquartile range (IQR). The IQR effect size is the change in response associated with a change in a predictor from the first quartile (Q1, 25th percentile) to the third quartile (Q3, 75th percentile), which includes 50% of the data values

The predictive models S and E are illustrated in Figs. 2 and 3. The cumulative fit plots display predicted probabilities for each individual by accumulating the contribution of one predictor at a time. A dotted black “trend line” connects the mean cumulative value at each predictor. Each orange path represents a patient with IBD, while each green path represents a control. The thick black vertical line near the righthand edge of the graph indicates the cumulative predicted probability of having IBD, summing up the effects for all of the predictors. The effect size tables show the interquartile range (IQR) or overall range effect sizes for each predictor, with predictors ordered by the effect size. The IQR effect size is the odds ratio associated with a change in a predictor from the first quartile (Q1, 25th percentile) to the third quartile (Q3, 75th percentile), which includes 50% of the data values. The effect size is expressed in terms of overall range when the Q1 and Q3 values are equal. The S model (Fig. 2, AUC = 0.93) has 7 predictors, including age and fecal calprotectin. The model also includes metabolites and microbes. A person with a relative abundance of *Subdoligranulum* of 0.07 is approximately half as likely to have IBD as a person with no *Subdoligranulum*, holding all other variables constant. The E model (Fig. 3, AUC = 0.97) has 13 predictors, and includes enzymes, metabolites, and microbes. A person with 6-phosphofructokinase of 310 ppm is approximately 5.7 times more likely to have IBD than a person with 236 ppm, holding all other variables constant.

**Fig. 4:**
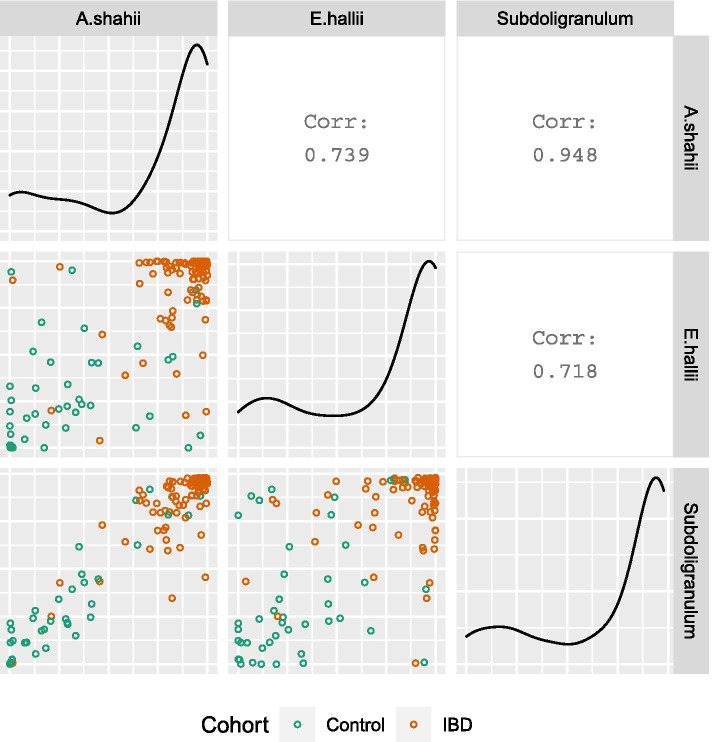
Comparison of predicted probabilities from top 3 CANTARE models. Predictions from 3 logistic regressions, each seeded with the network neighborhood of a different microbe. Predictors in Model S are a proper subset of the predictors in Model A. The predictions from these models are more similar to each other (r = 0.948) than they are to the E model (r = 0.739 and r = 0.719), with which they have no overlapping predictors. Black lines in the cells on the diagonal represent the distributions of the predictions

Figure 4 compares predictions from the top 3 models. The S model is a proper subset of the A model, with all of the nodes in S also included in A. Their predictions from the associated models are more similar to each other (Pearson correlation ~ 0.9) than they are to the E model (Pearson correlations ~ 0.7), with which they have no overlapping predictors.

### Analysis of CANTARE configuration parameters

**Fig. 5:**
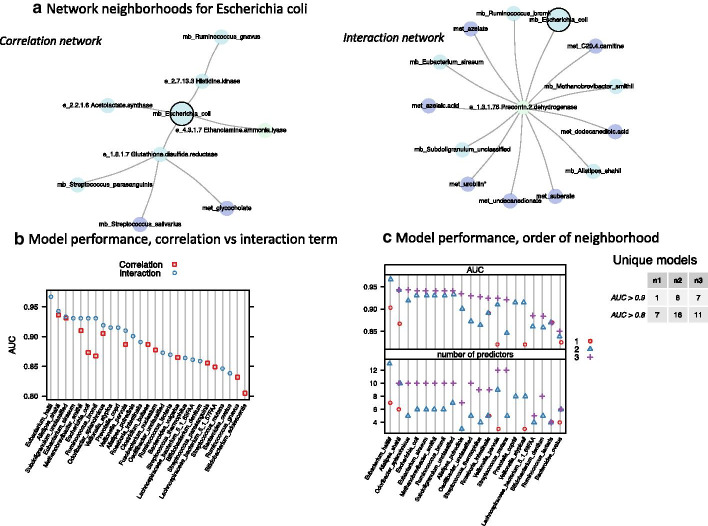
Comparison of model performance for differing configuration parameters. **a** Network neighborhoods for *Escherichia_coli* (highlighted with black border) from Vnet created using correlation (no interaction term) and Vnet using an interaction term. The two neighborhoods include different predictors, illustrating that the two Vnets include different analytes and relationships among analytes. **b** Comparison of model performance: Vnet created using correlation (no interaction term) compared to Vnet using an interaction term. The set of models for each configuration differs because the microbes in each Vnet differ. AUC for all models from each configuration is shown, with models ordered by AUC. When a model can be generated from both Vnets, the AUC for the interaction network is higher (mean = 0.93) than for the correlation network (mean = 0.90, p-value = 0.016). **c** Comparison of models by order of network neighborhood. AUC and number of predictors are shown for models generated using neighborhoods of order 1, 2, and 3. Models are ordered by maximum AUC, regardless of neighborhood order. For neighborhoods of order 3, models indicated by # (Eubacterium_hallii, Veillonella_atypica, and Prevotella_copri) failed to converge. Thus, no data exists for order 3 for these microbes. The number of unique models for AUC > 0.9 and AUC > 0.8 is shown in the accompanying table. Jitter has been added to minimize overplotting, but some symbols still overlap, such as AUC and the number of predictors for *Ruminococcus_lactaris* (second from right)

One important decision in the VOLARE-CANTARE workflow is the specification of the initial regression model for pairwise cross-omic comparisons. In this case study we specified a regression model with an interaction term to allow different relationships by IBD group (Fig. 1b). To assess the impact of this decision, we reapplied the workflow, starting with a simple correlation model between analytes. Again, we selected the top 35 relationships per assay pair, based on slope p-value (which is equivalent to the p-value from a Pearson’s correlation). The Vnet built from the pairwise correlations includes different predictors than does the Vnet built from the regressions with interaction terms. Thus, the underlying predictive models from the two Vnets include different predictors. Importantly, while we refer to models by the name of the microbe that was the hub of the neighborhood that seeded the model, the actual predictors in the neighborhoods generated by the two approaches generally differ. Figure 5 compares the model performance for the differing configurations. The correlation Vnet included 93 nodes, as compared to the 108 nodes of the interaction-term Vnet, with 37 in common. With respect to microbes, the correlation Vnet included 22 and the interaction-term Vnet 30, with 10 in common. Figure 5a highlights differences in the neighborhoods for *Escherichia_coli* for the two Vnets. We next generated predictive models for IBD group from microbe-centered network neighborhoods of order 2 (having at least 4 nodes), and compared the models generated by the two Vnets. In both configurations, all predictive models had AUC values greater than 0.8 (Fig. 5b). However, the interaction-term network had more models (20) than did the correlation network (14). As expected, not all microbes in the interaction-term network were in the correlation network. However, for microbes in both networks, the predictive model based on the interaction term was better, as defined by a higher AUC (p = 0.016, mean of interaction-term network = 0.93, mean of correlation network = 0.90).

Another configuration parameter is the order of the network neighborhoods that seed the CANTARE models. To illustrate the impact of this parameter, we built models based on neighborhood order of 1, 2, and 3, for neighborhoods with at least 4 nodes. Neighborhoods of order 1, 2, and 3 yielded 7, 16, and 11 unique models with AUC above 0.8, respectively (Fig. 5c). For neighborhoods of order 3, the estimation for 3 models failed to converge.

### Comparison to random forests and penalized regressions

To compare CANTARE to random forest classifiers, we generated three random forests from each of two different data sets: (1) all multi-omic data, plus age and fecal calprotectin; and (2) the analytes in the Vnet, plus age and fecal calprotectin. Hereafter, we refer to these as the “U” forests and the “V” forests. The 3 AUC values for the U forests ranged from 0.92 to 0.93, while the AUC values for the V forests ranged from 0.93 to 0.94. (Franzosa reported AUC values of 0.92, 0.90, and 0.92, respectively, for random forests trained on metabolites, microbial species, and a combination thereof [3].) For each forest, we used the percentage of times a sample was classified as IBD as a probabilistic measure of prediction. Forests starting with the same underlying data set had greater correlation of prediction probabilities with each other than across data sets (Additional file 2: Fig. S1).

Following the pattern established with random forests, we generated three penalized regressions using all the multi-omic data, plus age and fecal calprotectin (hereafter, the “U” regressions), and three regressions using the analytes in the Vnet, plus age and fecal calprotectin (hereafter, the “V” regressions). AUC for both the U and V penalized regressions ranged from 0.92 to 0.93. As with random forests, penalized regressions based on the same underlying data had greater correlation of predicted probabilities with each other than across data sets (Additional file 3: Fig. S2). Two of the three U regression models (U_1 and U_3) were duplicates of each other. In a separate analysis, the creation of 100 U regression models yielded 29 unique models. Thus, the duplication was not an aberration.

**Fig. 6:**
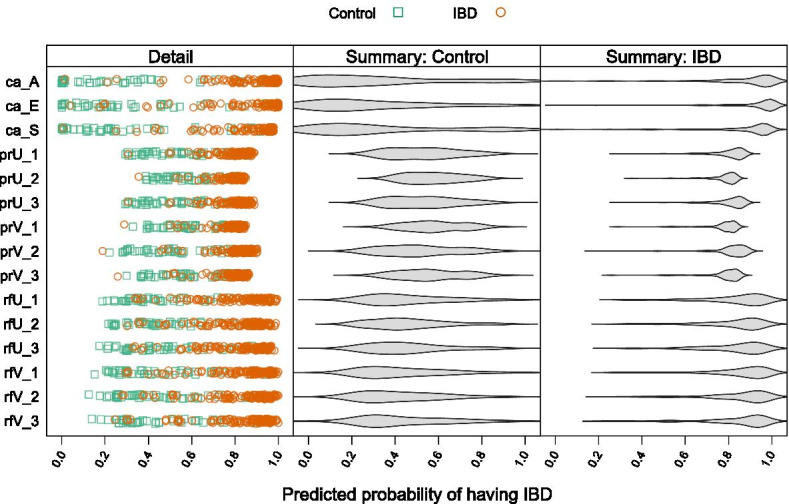
Distribution of predicted probabilities. Left panel: participant-level detail of predicted probabilities of having IBD for 3 CANTARE models, 6 penalized regressions, and 6 random forests. Green squares represent controls, orange circles represent people with IBD. Predictions made by CANTARE models have greater dynamic range than those made by penalized regressions or random forest (p-value = 1.3 × 10^–05^). Predictions made by CANTARE models have greater separation between high and low values. Center and right panels: violin plots illustrating overall distribution of predicted probabilities, separated by IBD group

Figure 6 illustrates the distribution of predictions across the top 3 CANTARE models, the 6 penalized regressions, and the 6 random forests. The range of predicted probabilities is greater for the CANTARE models than for the other methods (p-value = 1.35 × 10^–5^). Furthermore, participant-level detail of predicted probabilities for the CANTARE models show clear separation between the lower and higher values.

**Fig. 7:**
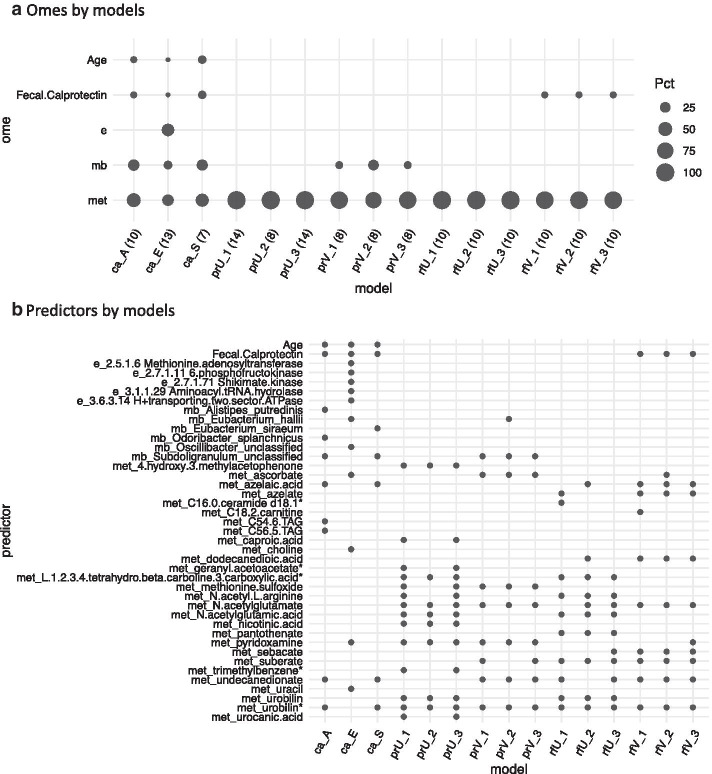
Comparison of predictors. **a** Omes by model, with c = CANTARE, p = penalized regression, r = random forest. The number of predictors included is shown in parentheses after the model name. Each circle represents the inclusion of age, fecal calprotectin, or any of 3 omes (e = enzyme, mb = microbe, met = metabolite) in each of 15 models. For random forests, the ten most important variables are considered. Circle size represents the percent of predictors/important variables drawn from that ome. **b** Included predictors/important variables by model. All variables that are included as predictors or identified as important are listed. Circles indicate which predictors are included in which models. Predictors flagged with an asterisk (*) are metabolic isomers. After age and calprotectin, variables are listed alphabetically, within ome

We also compared the presence of the three omes across all three methods (Fig. 7a). The only ome included in the U regressions and random forests was the metabolome. Furthermore, all models included metabolomic features. Microbiome features were included in both CANTARE models and V regressions. CANTARE model E included gene expression. Across the 8 unique CANTARE models with AUC > 0.90 (data not shown), the mean number of omes was 2.25. This is significantly different (p-value = 0.005) than the mean number of omes across the 12 forests and penalized regressions (mean = 1.25). Figure 7b tabulates which analytes were included as predictors (or important, in the case of random forests). For random forests, the list includes the 10 most important variables for each predictor. Only the CANTARE models included age, while the CANTARE models and the V forests included calprotectin. All models except CANTARE E included an isomer of urobilin (indicated by “urobilin*”). The CANTARE E model could not include this isomer because it was not in the original set of predictors as defined by the network neighborhood.

**Fig.8:**
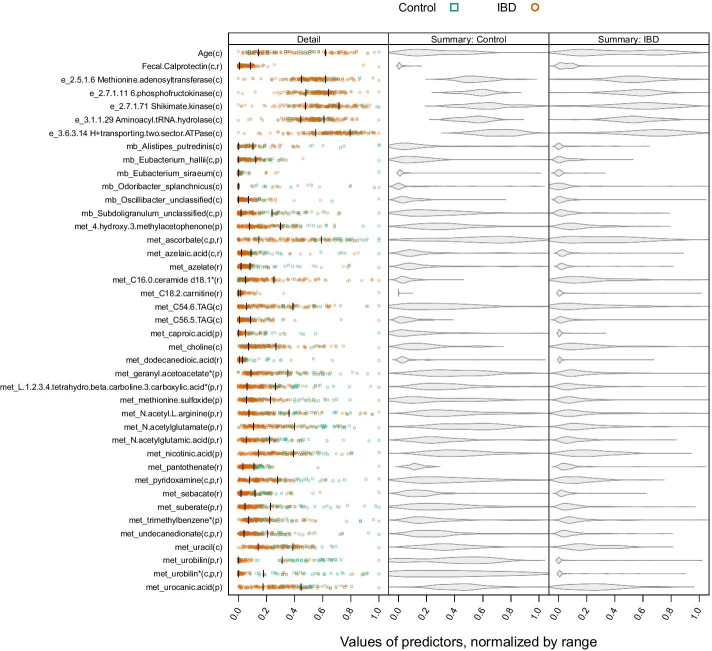
Distributions of predictors. Left panel: participant-level detail of predictors. Predictors across all models were normalized by range. Green squares represent controls, orange circles represent people with IBD. Jitter is used to reduce overplotting. Vertical lines indicate first and third quartile of predictor. Half of the observed values fall between these lines. *Eubacterium siraeum* and *Oscilbacter splanchnicus* are zero-heavy, with at least half of the observations being zero. Predictors flagged with an asterisk (*) are metabolic isomers. Parenthetical values after predictor names indicate which group of methods included the predictor, with c = CANTARE, p = penalized regression, r = random forest. After age and calprotectin, variables are listed alphabetically, within ome. Center and right panels: violin plots illustrating overall distribution of predictors, separated by IBD group

Figure 8 illustrates the distributions of the predictors, normalized to range, showing both participant-level detail and overall shape of the distributions by IBD group. Compared to the other omes, the enzymes have a long left tail. In contrast, the metabolites tend to have a long right tail, with several (met_azelaic.acid, met_azelate, met_C18.2.carnitine, met_C56.5.TAG, met_caproic.acid, met_dodecanedioic.acid) having 75% of their observations in the lowest 10% of the range. The microbes *Eubacterium siraeum* and *Oscilbacter splanchnicus* are zero-heavy, with at least half of the observations being zero.

## Discussion

We have presented a novel workflow for generating interpretable multi-omic models. We illustrated the approach in an IBD case study. We analyzed the impact of configuration parameters, and compared CANTARE models to those generated by random forests and elastic net penalized regression. CANTARE models are competitive with random forests and elastic net, and more likely to include features from more omes. In this section, we discuss the biomedical implications of the best-performing model, provide additional context to the VOLARE-CANTARE workflow, and elaborate on the comparisons to random forests and elastic net. We do not claim that our methodology is inherently better than these alternatives. Rather, we claim that it offers a novel approach for generating interpretable multi-omic models. We discuss limitations and strengths of our approach and conclude with selected thoughts about future work.

### Biomedical interpretation of Eubacterium hallii model (Model E)

To demonstrate that CANTARE models are parsimonious and interpretable, supporting further investigation, we offer the following discussion of Model E, the best performing in terms of AUC. The 13 predictors include 2 gut microbes, 5 microbial enzymes, 4 metabolites, age, and calprotectin. This model includes predictors that are protective of IBD (OR < 1) or increase the risk for IBD (OR > 1). In the following, all odds ratios are expressed in terms of changes from the 25^th^ percentile to the 75^th^ percentile (IQR). The 2 gut microbes, *Eubacterium hallii* (OR = 0.19) and *Oscillibacter unclassified* (OR = 0.64), are well described in the literature, as abundant members of a healthy gut microbiome that are often found in decreased abundance in IBD [38–40]. These identified microbes may be targets for modifying the IBD microbiome composition towards a healthier gut through diet [38]. Additionally the small molecules ascorbate (Vitamin C) (OR = 0.14), pyridoxamine (Vitamin B6) (OR = 0.16), and choline (OR = 0.15), which are found in various fruits, vegetables, and grains have been implicated in decreasing inflammation in IBD, and therefore are potential dietary modifiers that can be used towards nutritional strategies to prevent and treat IBD [41–43].

Shikimate kinase (OR = 0.10) operates within the shikimate pathway found in plants and bacteria and contributes to assembly of the basic building blocks for the range of aromatic metabolites and aromatic amino acids [44]. Metabolites that are derived from aromatic compounds provide ultraviolet protection, electron transport, and signaling molecules, and they serve as antibacterial agents which are beneficial to gut health [44]. In the diet, the presence of glyphosate from genetically modified agricultural environments disables Shikimate kinase and may result in the imbalances of the gut bacteria in IBD [45].

In clinical practice, fecal calprotectin is a highly sensitive biomarker that is routinely used as a marker of endoscopically active IBD [46]. In the model, higher levels of fecal calprotectin are expectedly associated with higher risk of IBD (OR = 19.83), but interestingly the enzyme methionine adenosyltransferase (MAT) has a higher predictive value (OR = 38.24). MAT genes encode enzymes responsible for the biosynthesis of S-adenosylmethionine, the principal biological methyl donor and precursor of polyamines and glutathione [47]. There is increasing evidence suggesting that MATs play significant roles in the development of cancers [47]. IBD patients have chronic inflammation which is an underlying risk factor for colon cancer, and mouse models demonstrate that tumor necrosis factor α (TNF-α), a target of IBD treatments, plays a critical role in development of inflammation-induced colon cancer [48–50]. MAT may be a novel biomarker with higher sensitivity than calprotectin that has not yet been studied in IBD patients. Two enzyme predictors of IBD, aminoacyl tRNA hydrolase (OR = 8.06) and phosphofructokinase (OR = 5.69), are observed in the literature related nonspecifically to pharmacotherapy and microbial metabolism, which may be related to IBD treatment and commonly associated microbes [51]. Although the model indicates the enzyme H + transporting two sector ATPase (OR = 0.01) is a strong predictor for protection, there is no clear role for it in IBD.

IBD is a complex disease with heterogenous clinical outcomes. The CANTARE models include features associated with reduced and increased odds of IBD. As such, these models may provide valuable insights for identifying non-invasive clinical biomarkers for therapeutic intervention to IBD patients. These features may also aid in characterizing IBD pathogenesis, etiology, and diagnosis. Though our discussions of biological relevance are speculative, the identified predictors provide the foundation for testable hypotheses in future studies.

### CANTARE workflow and parameter considerations

At a more general level, this IBD case study demonstrates that CANTARE supports multi-omic hypothesis generation; identifies quantitative, scorable relationships among a handful of analytes and an outcome of interest, with analytes drawn from multiple omes; delivers individual-level visualization of these relationships, for both the pairwise cross-omic relationships and multi-variable predictive models; and provides flexibility in both the building of the Vnet and the specification of the predictive models by leveraging existing regression machinery. For example, the pairwise cross-omic regressions of the Vnet used linear regression with an interaction term for IBD group, while the CANTARE model used logistic regression to predict IBD. Our primary goal in formulating the pairwise cross-omic regressions is to generate straightforward relationships between the analytes that can be easily visualized and vetted in units of measure that are familiar to biologists. In some cases, it might be appropriate to incorporate other covariates into these models, and present the pairwise regressions as partial correlation plots of residuals. Such residuals would represent a transformation of the underlying data, upstream of the VOLARE workflow.

The Vnet provides a multi-omic scaffold for the CANTARE models. In this case study, it includes the same number of edges for each of the cross-omic comparisons (enzymes to microbes, microbes to metabolites, and metabolites to enzymes). All but one of the 20 neighborhoods, from which CANTARE models were built, spanned all the three omes. While it is possible that any particular Vnet contains only two-node subnetworks with no additional connecting edges, we have not yet observed such a network. Biologically, highly connected networks, informally called “hairballs,” seem more common than sparse networks [52]. In addition, non-neighborhood variables of interest can be included in the CANTARE models. In the IBD case study, we included age and calprotectin. Other predictors of known relevance, whether omic or not, could also be included. In a fine-tuning step, predictors from disjoint neighborhoods (e.g. S and E, Fig. 1d) could be combined.

Turning to algorithmic details, we examined the impact of CANTARE configuration parameters—both the configuration of the Vnet and the order of the network neighborhood for predictive models. The predictive models built from a Vnet that accounted for IBD with an interaction term performed better than did those built from a Vnet that did not account for IBD. We reason that the better performing models were seeded with underlying information about IBD group. That said, the predictive models built from the correlation Vnet might provide different insights, since the underlying relationships represent strong correlations between analytes, independent of IBD. As expected, increasing the order (and thus number of nodes) of the network neighborhood introduces a tradeoff between model performance and number of predictors. In general, the larger networks have better performance at the cost of a larger less-parsimonious model. Neighborhoods of order 3 yielded fewer unique models than neighborhoods of order 2 (11 versus 16, with AUC > 0.8), due to overlapping neighborhoods. If the edge cut for the Vnet were more permissive (e.g. 50 per assay pair instead of 35 per assay pair), it is likely that there would be more unique models with increasing neighborhood order. Also, in a few cases, the model estimation failed to converge*.* Our case study included 153 participants. As the number of samples increases, more predictors can be supported. For example, Harrell suggests 10 to 15 events per simple predictor in logistic regression models [53]. Thus, study size may influence the appropriate neighborhood order.

### Comparison to random forests and penalized regressions

We also compared CANTARE models to penalized regressions and random forests, considering overall performance (as measured by AUC), sample-level predictions, and the predictors themselves. The AUC values of CANTARE models were comparable to those of random forests and penalized regressions, whether the forests or regressions were generated with the universe of multi-omic data or the data underlying the Vnet. The dynamic range of the predicted probabilities of the CANTARE models was greater than that of random forests and penalized regressions. The CANTARE models and the V penalized regression models included predictors from at least two omes, with model E and two others (hubs of *Prevotella_copri* and *Veillonella_parvula*) having predictors from all three omes.

The most important variables in all six random forests included only metabolites. Metabolites account for 58% of the analytes in the U forests and 50% of the analytes in the V forests. These proportions are not so large as to make metabolite-only forests probable. Thus, there may be aspects of the distribution of metabolites that make them conducive to selection by random forests. Microbes are included in only the CANTARE models and V regressions. The zero-heavy distribution of many species may make them less likely to be important predictors for random forests and penalized regressions. In the case of penalized regression, the absolute value of each regression coefficient contributes to the overall constraint. Thus, a predictor that provides additional model accuracy for only a handful of samples may be suboptimal. For example, *Odoribacter splanchnicus*, which is a predictor in model A, has only 21 non-zero values (11 control, 10 IBD). Including this predictor in a penalized regression would impact the model for these 21 samples only.

Both random forests and penalized regressions have random components that result in slightly different important variables or predictors on repeated runs. The random components in forests include the selection of samples for building each tree, and the subset of variables that are considered at each split of the tree. The random component of penalized regression is in the cross-validated estimation of lambda, a constraint on the model coefficients. The glmnet package offers a function for automatically selecting lambda as the largest value that is within one standard error of the value which minimizes error. While these random components aim to reduce overfitting, analysis of any single model can only be considered representative. CANTARE, in contrast, is deterministic, although susceptible to overfitting.

### Limitations

There are limitations to this work. First, we provide only one case study. However, we examined the effect of configuration parameters and compared a variety of CANTARE models to random forests and penalized regression, considering overall performance, predicted probabilities, and included predictors. These analyses place CANTARE in context with other algorithms. In other work [27], we applied the method to continuous cardio-metabolic outcomes associated with a short-term weight loss intervention. Second, the current implementation is a collection of R scripts accessible to analysts who have intermediate R skills and are comfortable formulating regression models and dissecting the result objects. While this may be a barrier to potential users, it does allow flexibility in specification of the pairwise regressions and the predictive models. Third, we cannot claim that our models are optimal. There may well be other multi-omic parsimonious models with superior performance. Fourth, as regression models, the CANTARE models are subject to the general constraints of linear regressions, such as linearity with log odds or continuous outcomes, normal distribution of the errors, and little to no multicollinearity between predictors [53]. Fifth, while the CANTARE models provide a consolidated quantitative framework for multiple omic predictors, they do not provide a mechanistic framework suggesting a sequence of linked events (e.g. *Bacteroides fragilis* interacts with regulatory T cells which in turn produce IL-10 [15]).

### Strengths

The CANTARE workflow offers a number of strengths. First, the CANTARE models are parsimonious and interpretable. They identify a handful of predictors that collectively support conversation, further investigation in the literature, and follow-up experiments. Second, this handful of predictors is multi-omic by design. The Vnet consists of edges that encapsulate relationships across omes, with a similar number of edges for each pair of omes. The CANTARE models are seeded with subsets of this Vnet. Third, the relationships between the predictors and the outcome are quantitative and directional. We can identify which predictors have the largest effect sizes, and whether they are advantageous or disadvantageous to the outcomes. Fourth, the workflow is customizable and allows the analyst to leverage a variety of regression models such as linear regression, logistic regression, and mixed effects models. Fifth, because they are regression models, the CANTARE models can be scored by well-established techniques such as AUC or mean-squared error. These scoring techniques support the evaluation of workflow configuration. Sixth, the workflow is supported by visual representations of key steps. Both the pairwise regression plots and the cumulative fit plots of the CANTARE models illustrate person-level patterns. Seventh, given a particular set of tuning decisions, the results are deterministic.

### Future work

The analysis presented herein suggests several areas of future work. First, different types of regressions can be used in both the VOLARE and CANTARE components of the method. For example, quadratic or cubic terms could be added to the pairwise regressions to identify non-linear relationships. Significant terms could then be incorporated into predictive models. Second, an effort to better understand why certain methods select predictors from certain omes is warranted. This future work could consider the impact of group balance within data sets, and might include an analysis of the interactions and functional forms of relationships between predictors selected by random forests. Such work could influence both upstream data transformations specific to particular omes, and strategies for making algorithms more ome aware. Third, interactive visualization of multi-omic models could aid in their interpretation. For example, combining cumulative fit plots (Figs. 2, 3) with model-specific data distributions similar to Fig. 8 with synchronized brushing would allow an analyst to select outliers for one predictor and identify both the cumulative fit paths of the associated samples and the location of the selected samples within the distributions of other predictors. This would likely facilitate the identification of subgroups within the context of the predictive model.

## Conclusion

We described the CANTARE workflow and applied it in an IBD case study, generating 8 unique multi-omic predictive models with an AUC over 0.9. We provided a detailed discussion of the biological relevance of the best model, demonstrating interpretability. CANTARE models are competitive with those generated by random forests and penalized regressions (as measured by AUC), with CANTARE predictions showing a larger dynamic range (p-value = 1.35 × 10^–5^). CANTARE models are more likely to include predictors drawn from more omes. CANTARE can be applied to a variety of experimental designs, supporting hypothesis generation for follow-up investigations.

## Supplementary Information

**Additional file 1.** Multi-omic data analyzed.

**Additional file 2.** Comparison of predicted probabilities from random forests.

**Additional file 3.** Comparison of predicted probabilities from penalized regressions.

## Data Availability

All data generated or analyzed during this study are included in this published article and its supplementary information files.

## Abbreviations

AIC: Akaike information criterion
AUC: Area under the curve
CANTARE: Consolidated Analysis of Network Topology And Regression Elements
CD: Crohn’s disease
CV: Coefficient of variation
DFFITS: Standardized difference in fit
EC: Enzyme Commission
GI: Gastrointestinal
IBD: Inflammatory bowel disease
IQR: Interquartile range
JSON: JavaScript Object Notation
LC/MS: Liquid chromatography/mass spectrometry
MAT: Methionine adenosyltransferase
OR: Odds ratio
UC: Ulcerative colitis
Vnet: VOLARE network
VOLARE: Visual analysis of LineAr Regression Elements
