# Supplementary material for: CANTARE: finding and visualizing network-based multi-omic predictive models
Source: BMC Bioinformatics. 2021 Feb 19;22:80. doi: 10.1186/s12859-021-04016-8 (PMC7896366; doi:10.1186/s12859-021-04016-8)

Supplemental Figure 1 : Comparison of RF predicted probabilities for U and V forests

V forests are more similar to each other than to the U forests, while U forests are more similar to each other than to the V forests. rfU = random forest generated from the "universe" of multi-omic data. rfV = random forest generated from the Vnet.

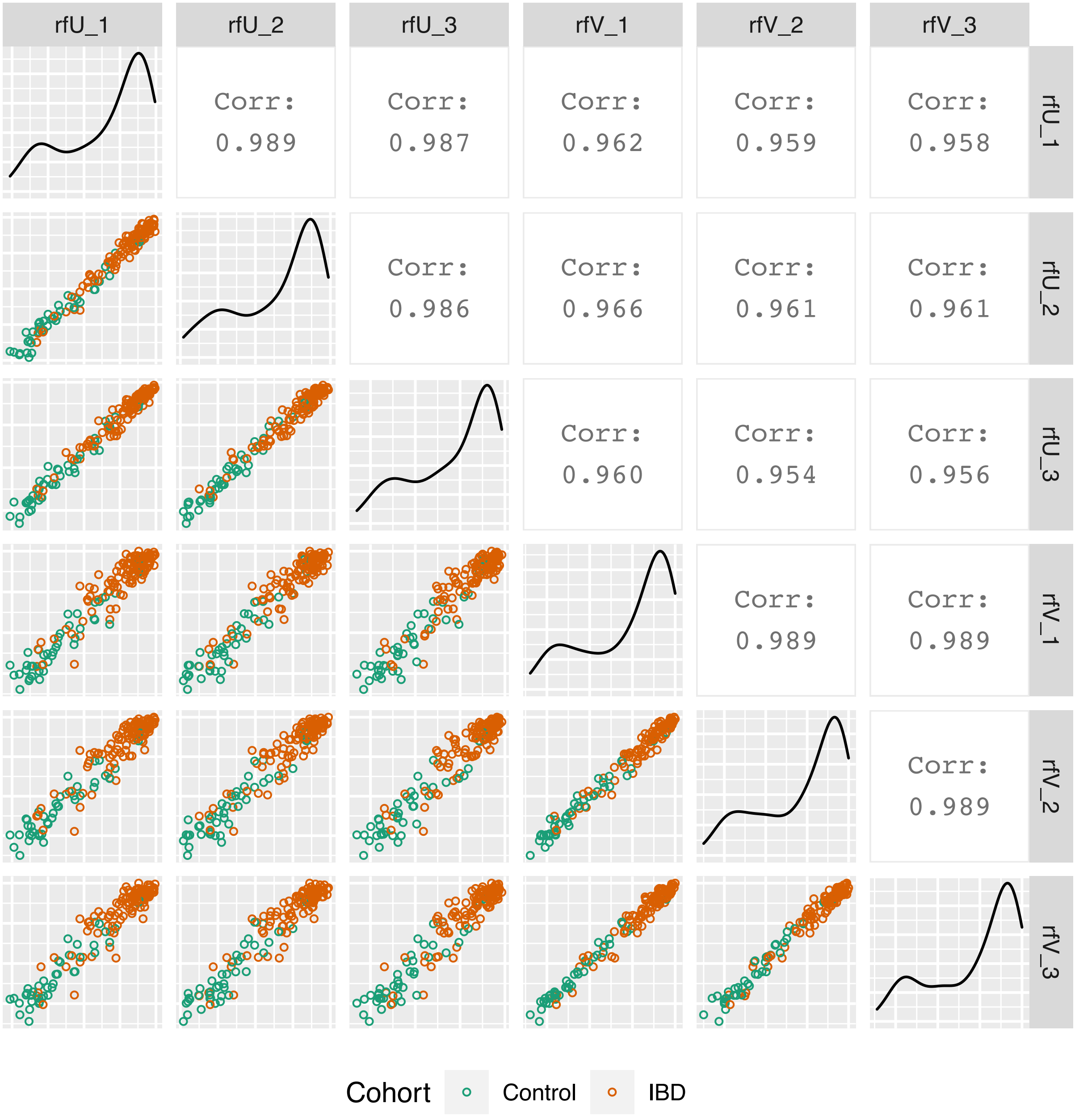

Supplement: Supplementary file 2 — Additional file 2. Comparison of predicted probabilities from random forests. [file 12859_2021_4016_MOESM2_ESM.pdf]
