# Supplementary material for: CANTARE: finding and visualizing network-based multi-omic predictive models
Source: BMC Bioinformatics. 2021 Feb 19;22:80. doi: 10.1186/s12859-021-04016-8 (PMC7896366; doi:10.1186/s12859-021-04016-8)

Supplemental Figure 2: Comparison of predicted probabilities for U and V penalized regressions

V regressions are more similar to each other than to the U regressions, while U regressions are more similar to each other than to the V regressions. prU = penalized regressiongenerated from the ”universe” of multi-omic data. prV = penalized regression generated from the Vnet. The models, prU_1 and prU_3 were duplicates of each other. In a separate analysis, the creation of 100 U regression models yielded 29 unique models. Thus, the duplication was not an aberration.

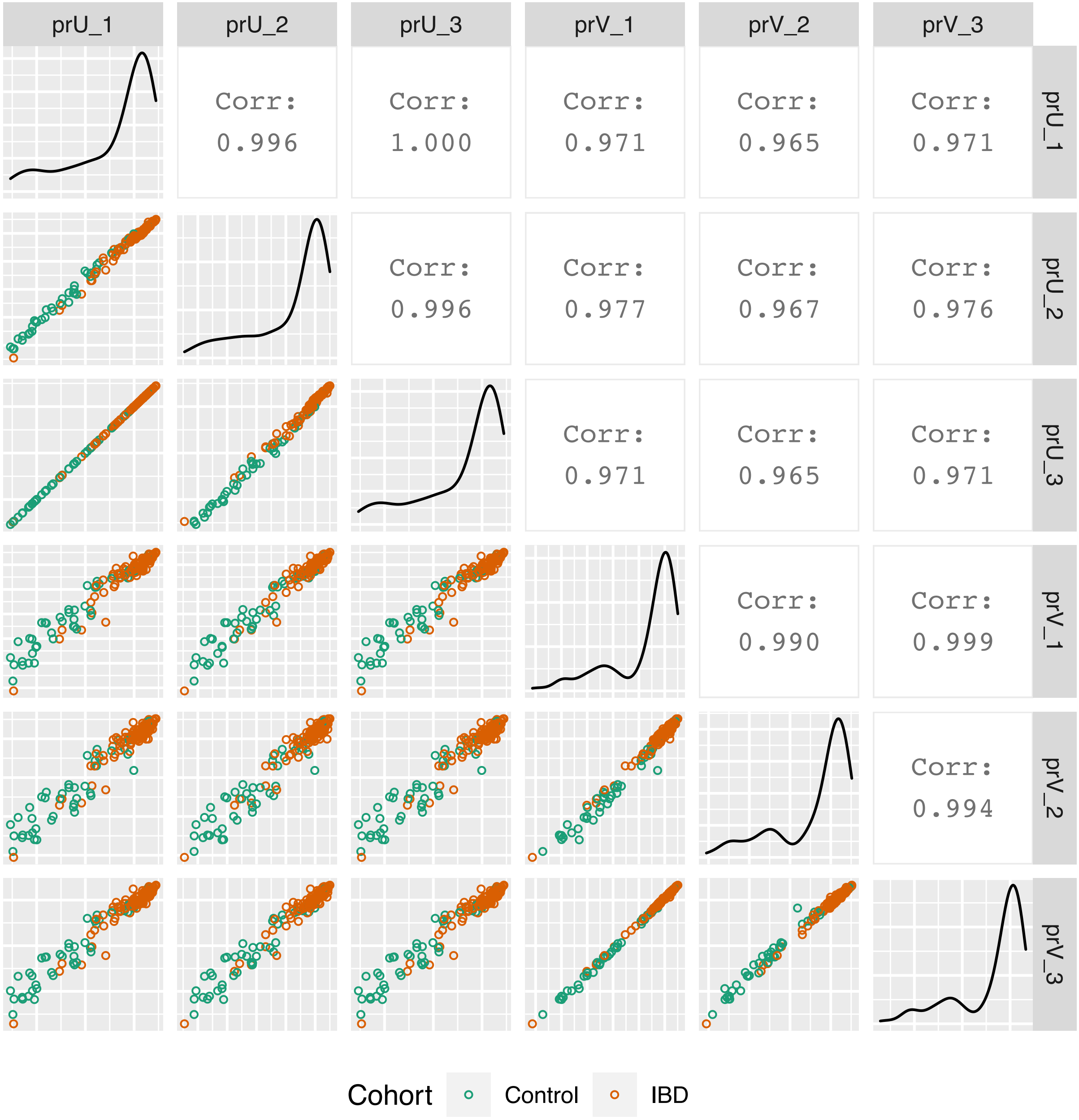

Supplement: Supplementary file 3 — Additional file 3. Comparison of predicted probabilities from penalized regressions. [file 12859_2021_4016_MOESM3_ESM.pdf]
